# Exploring the Antimicrobial Potential of Natural Zeolites for Cleaner Water and Air: A Comprehensive Review

**DOI:** 10.3390/ma19214707

**Published:** 2026-11-05

**Authors:** Francesca Gattucci, Mari Sofia Lallukka, Marta Miola

**Affiliations:** Department of Applied Science and Technology, Politecnico di Torino, Corso Duca degli Abruzzi 24, 10129 Turin, Italy; francesca.gattucci@polito.it (F.G.); mari.lallukka@gmail.com (M.S.L.)

**Keywords:** natural zeolites, antimicrobial, environmental remediation, air filtration, water purification

## Abstract

Microbial contamination of air and water remains a major global health and environmental challenge, while scalable and sustainable antimicrobial materials are still insufficiently developed. Natural zeolites represent a promising green platform owing to their abundance, low cost, chemical and thermal stability, structural porosity, and intrinsic ion-exchange capacity, which enable their functionalization with a wide range of antimicrobial agents. This review examines the antimicrobial potential of natural zeolites, focusing on the structural and chemical features that promote the incorporation of metal ions (Ag^+^, Cu^2+^, Zn^2+^, Co^2+^), nanoparticles (Ag, CuO, ZnO, TiO_2_, Au, NiO), and antimicrobial molecules. The main microbial inactivation mechanisms, including oxidative stress, membrane disruption, enzyme inhibition, and ion release, are discussed. Applications in both air and water treatment are critically reviewed, including VOC adsorption, particulate matter removal, airborne pathogen inactivation, and the removal of heavy metals, ammonium, organic micropollutants, radiological species, and microbial contaminants from water. Finally, current challenges in translating zeolite-based antimicrobial systems into scalable real-world technologies are highlighted, together with future directions toward sustainable and multifunctional treatment solutions.

## 1. Introduction

Clean air and safe water are essential for human health, yet both are increasingly under threat due to the consequences of industrialization and urban growth. As urban lifestyles have evolved, people now spend nearly 90% of their time indoors, often in enclosed spaces where air circulation is limited [1]. At the same time, industrial operations, farming activities, and urban sprawl have severely affected water ecosystems such as rivers and oceans, compromising their quality and threatening ecological and human well-being [2].

According to the World Health Organization (WHO), approximately 69% of diarrhoeal deaths worldwide in 2019 were attributable to unsafe water, sanitation, and hygiene (WASH) services, accounting for over one million deaths [3].

Over fifty distinct diseases are associated with poor water quality, highlighting the urgent need for improved treatment technologies [2].

In indoor environments, air quality is diminished by both chemical and biological pollutants. Chemical agents such as nitrogen oxides (NOx), sulfur dioxide (SO_2_), ozone (O_3_), volatile organic compounds (VOCs), particulate matter (PM), radon, and heavy metals are commonly identified contaminants [4]. Equally problematic are biological pollutants, including viruses, bacteria, fungi, dust mites, pollen, pet dander, and insects [5]. Indoor air pollution (IAP) has become a risk to public health and is often more dangerous than outdoor pollution [1].

Microbial contamination inside buildings primarily stems from human respiration, through talking, coughing, breathing, and sneezing, which releases aerosolized particles containing bacteria and viruses. Pets further increase microbial load via skin, saliva, excreta, and parasites. Environmental parameters like moisture, humidity, and temperature also regulate microbial presence indoors. Poorly designed buildings and inadequate airflow contribute to microbial growth, and HVAC (Heating, ventilation, and air conditioning) systems, when not regularly maintained, become hotspots for bioaerosols. Damp filters, condensation, and clogged vents can promote the spread of molds and bacteria throughout indoor spaces [5].

The health effects of microbial air contamination are significant, particularly in relation to respiratory infections. Disinfection technologies capable of removing airborne pathogens, such as those associated with SARS and COVID-19, are especially vital in clinical settings. Common air purification methods include HEPA filters, ultraviolet light, and electrostatic systems, though many of these face technical and operational challenges [6].

Water quality issues also arise from sources like industrial waste, agricultural runoff, and insufficient wastewater treatment. Key polluters include sectors such as pulp and paper, textiles, distilleries, food processing, tanneries, and the metallurgical and nuclear industries. Additionally, untreated sewage and natural contaminants further degrade water sources [7].

To address the shortcomings of conventional purification methods, antimicrobial zeolites have gained attention as an innovative and eco-compatible alternative for air and water treatment. Both natural and synthetic forms have demonstrated the ability to remove various pollutants, making them promising materials for environmental remediation [8].

Despite the growing number of review papers on antimicrobial zeolites, most studies primarily focus on synthetic systems or emphasize antimicrobial efficiency without explicitly addressing the role of natural zeolites and their structure–performance relationships. While synthetic zeolites offer high tunability and structural control [9], natural zeolites are particularly attractive due to their wide availability, low cost, and suitability for large-scale applications, yet these aspects are often underexplored in the literature.

To address this gap, the present review focuses on natural zeolites, exploring their functionalization strategies for the development of effective antibacterial filtration materials and examining the relationship between their physicochemical properties, antimicrobial activity, and suitability for air and water treatment applications. This combined perspective provides a comprehensive overview of the factors governing the performance of natural zeolite-based antimicrobial materials while highlighting unresolved challenges and future research directions.

## 2. Properties of Natural Zeolites

Zeolites are crystalline materials constructed from tetrahedral units composed of aluminum (Al), silicon (Si), or phosphorus (P), each coordinated to four oxygen atoms. These units interconnect through shared oxygen, forming a three-dimensional framework. The linkage of these primary tetrahedra through oxygen bridges gives rise to more complex structures, such as rings and cavities, which define the porous nature of the zeolite [10].

Their unique structure allows zeolites to act as “molecular sieves,” selectively interacting with specific molecules based on size and charge [11]. This selectivity arises from negatively charged [AlO_4_]^−^ units in the framework, which are balanced by mobile cations, commonly Na^+^, K^+^, or Ca^2+^, residing within the pores. These cations, along with water molecules, are not rigidly fixed and can migrate through the structure, enabling ion exchange and reversible dehydration. As such, the properties of zeolites depend on the size, connectivity, and chemical makeup of their pores [12].

According to IUPAC classification, zeolites are considered microporous if their pores are smaller than 2 nanometers, while those with pores from 2 to 50 nanometers fall into the mesoporous category [13].

Zeolites occur in both natural and synthetic forms. Natural zeolites, such as clinoptilolite, mordenite, and chabazite, are derived from volcanic or sedimentary sources [14]. Significant natural reserves are found in geologically active areas with past volcanic activity [15]. Synthetic zeolites, in contrast, are fabricated by heating raw materials like feldspar, China clay, or soda ash under controlled conditions that replicate geological processes. This synthetic route offers greater precision in pore architecture but comes with higher costs. Despite their abundance and affordability, natural zeolites often contain mineral impurities that reduce crystallinity and limit their industrial use. Techniques like acid leaching are commonly applied to improve purity, increase the Si/Al ratio, and enhance their adsorption capabilities [16].

Zeolite materials can be engineered using either bottom-up or top-down approaches to introduce mesoporosity. In bottom-up synthesis, mesopores are integrated directly during crystal formation using templating or template-free strategies. In contrast, top-down methods involve modifying existing crystals through treatments like demetallation or exfoliation to create additional porosity [10].

Thanks to their high internal surface area, structural tunability, and ion-exchange properties, zeolites have a broad spectrum of applications. These include water purification, gas separation, membrane filtration, coagulation, and antimicrobial action. Their effectiveness in such roles stems from a combination of uniform pore size, mobile framework cations, and adjustable surface polarity, making them vital materials in environmental and industrial technologies [17].

When referring to natural zeolites, it is worth noting that the most relevant types for practical applications include clinoptilolite, mordenite, and chabazite, owing to their abundance and suitable physicochemical properties [18]. These materials can be further classified based on their framework Si/Al ratio, which varies depending on their geological origin. This parameter is particularly important because it determines the negative charge of the aluminosilicate framework, which is balanced by exchangeable cations such as Na^+^, K^+^, and Ca^2+^. These cations are mobile and can be exchanged with species present in the surrounding environment, enabling ion-exchange processes [19]. In general, zeolites with a Si/Al ratio higher than 4 exhibit a relatively low ion-exchange capacity, whereas materials with lower ratios are more prone to cation exchange [20]. Consequently, the Si/Al ratio can influence the amount of antimicrobial metal ions, such as Ag^+^, Cu^2+^, and Zn^2+^, that can be incorporated into the zeolitic structure [21]. However, the actual metal loading also depends on the type and accessibility of exchangeable cations.

In addition to ion-exchange behavior, the Si/Al ratio also influences the hydrophilic–hydrophobic character of the material and its chemical stability. Specifically, an increase in the Si/Al ratio leads to a more hydrophobic framework and enhanced stability under certain operating conditions [22].

Typical examples include natural chabazite, which exhibits a Si/Al ratio in the range of approximately 3–3.5 [23], while natural clinoptilolite and mordenite generally show higher ratios, often exceeding 4 [22,24].

Furthermore, natural zeolites may contain variable amounts of non-zeolitic mineral phases and impurities, depending on their geological origin. These components can affect the effective zeolite content and the accessibility of exchange sites, potentially influencing metal uptake and the reproducibility of functionalization processes. Consequently, pretreatment strategies, such as sodium enrichment or controlled chemical purification, may be employed to improve the availability of exchangeable sites and facilitate the incorporation of antimicrobial metal ions [25].

Although these structural parameters do not directly determine antimicrobial activity, they strongly influence key physicochemical properties such as ion-exchange capacity, ion mobility, and the loading and release of antimicrobial agents, which ultimately govern the antimicrobial performance of the material. In this context, clinoptilolite is the most widely investigated natural zeolite, owing to its favorable balance between availability, ion-exchange capacity, and structural stability.

## 3. Mechanisms of Antimicrobial Functionalization in Zeolites

Natural zeolites do not inherently possess antibacterial properties [26]; however, they are thermally and chemically stable [27], low-cost [28], non-toxic (in their non-fibrous form) [29], and environmentally friendly [30]. These characteristics make zeolites excellent candidates for designing antimicrobial materials by serving as supports for antibacterial agents such as metal ions, nanoparticles, or biocidal molecules. From an ion storage and release perspective, zeolites provide a unique platform. Their aluminosilicate framework functions as an ion-exchange medium, capable of storing various metallic ions [31], which plays a critical role in their modification, especially for the incorporation of nanoparticles [32].

### 3.1. Metal Ions

Among the metal ions, silver (Ag^+^) is the most well-known for its strong antibacterial properties [33]. However, due to the overuse of silver and the increasing resistance that bacteria are developing against it [34], recent research has focused on finding alternative ions, such as zinc (Zn^2+^) and copper (Cu^2+^) [35,36], as well as iron [37], gold [38], and cobalt [39]. Each of these metals operates through distinct yet overlapping antimicrobial mechanisms.

Silver ions disrupt bacterial cells by collapsing membrane potential, interfering with the electron transport chain (ETC), and binding to essential proteins and enzymes. They also penetrate the cell, damaging DNA and ribosomes, which hinders gene expression. These disruptions weaken cell integrity, leading to membrane detachment and structural changes. Additionally, silver ions stimulate respiration, interact with ETC enzymes, and release Fe^2+^ ions, triggering reactive oxygen species (ROS) production and oxidative damage. Their antibacterial effectiveness depends on factors such as bacterial type, silver ion concentration, nutrient availability, and the presence of chelating compounds [40].

Copper ions affect microbial cells through multiple mechanisms, disrupting membrane structure, damaging biomolecules and organelles, and interfering with metabolism and gene expression, ultimately causing cell death. This makes copper ions an effective fungicide and antimicrobial agent. However, the dominant mechanism of action remains unclear [41].

Zinc is essential for microorganisms and higher organisms, playing a key role in cellular reactions at low concentrations. Its levels are tightly regulated by transporters, making Zn^2+^ ions generally non-toxic to higher organisms. However, excess Zn^2+^ (above ~10^−4^ M) disrupts homeostasis, leading to cytotoxic effects in prokaryotes. Due to this, Zn^2+^ exhibits antimicrobial properties, acting as both an antibacterial and antifungal agent [42].

Heavy metals have long been used as antimicrobial agents and are still applied in coatings and medical devices. Their toxicity mechanisms vary by metal and bacterial type but often involve oxidative stress. Metals like iron, copper, and nickel enhance ROS generation via the Fenton reaction, damaging DNA, proteins, and lipids. In addition, some metal ions oxidize enzyme residues, leading to protein degradation and loss of enzymatic activity. Others, such as gallium, mimic essential cofactors, thereby disrupting enzyme functions. Metals like cadmium can trigger lipid peroxidation, compromising membrane stability, while others interfere with electron transport or cause proton leakage, collectively leading to microbial cell death [43].

Zeolites, with their crystalline aluminosilicate framework, are highly effective ion-exchange materials. Their structure consists of interconnected SiO_4_ and AlO_4_ tetrahedra, sharing oxygen atoms and forming a negatively charged lattice due to the substitution of trivalent aluminum for silicon [44]. This negative charge is balanced by loosely bound alkali or alkaline-earth metal cations (e.g., Na^+^, K^+^, Ca^2+^), which reside in the pores and channels of the zeolite but are not fixed in position. Instead, they are mobile and replaceable, making zeolites capable of exchanging ions with their surrounding environment [45].

This ion-exchange ability allows zeolites to store and release bioactive metal ions, such as silver (Ag^+^), copper (Cu^2+^), and zinc (Zn^2+^), which are known for their antimicrobial properties [46]. When these ions partially replace the native cations in the zeolite structure, the resulting materials exhibit promising antibacterial and antimicrobial activity, making them suitable for medical, environmental, and hygienic applications [45].

To enhance the ion-exchange efficiency, zeolites are often pre-treated with sodium ions (Na^+^). This process—known as Na-enrichment or Na-homoionization—is widely used because sodium is more weakly bound in zeolites like clinoptilolite compared to other naturally occurring cations such as calcium or potassium. As a result, sodium-enriched zeolites more readily exchange Na^+^ with desired antimicrobial cations from solution [47].

#### 3.1.1. Silver

The functionalization of zeolites with silver can be effectively achieved through a straightforward immersion technique, where the zeolite powder is soaked in a solution containing a silver precursor. This method has proven both simple and efficient, as demonstrated by several studies. For instance, natural clinoptilolite exchanged with silver achieved 100% bacterial removal within just 30 min [48], while Iranian zeolitic tuff treated similarly exhibited antibacterial efficacy against *Escherichia coli* comparable to that of synthetic silver-exchanged zeolite A [27]. Following immersion, the functionalized zeolite is typically recovered through filtration.

In addition to liquid-phase ion exchange, solid-state ion exchange has also been explored. This method involves mixing zeolites with solid silver salts and subjecting the mixture to controlled heating. Notably, this approach has demonstrated the potential to achieve higher silver loading compared to conventional liquid-phase methods and has shown impressive antibacterial properties, especially against Gram positive bacteria, depending on the Ag precursor used [49,50].

To further enhance ion-exchange efficiency—particularly for silver ions—zeolites are often pretreated with sodium (Na^+^). This involves immersing the zeolite in a concentrated NaCl solution, converting it to its sodium form. The Na^+^ form significantly improves the zeolite’s capacity to exchange with silver ions, as Na^+^ is more readily displaced than naturally occurring cations such as Ca^2+^ or K^+^. Numerous studies have confirmed the benefits of this pretreatment in boosting metal uptake. For example, Mexican natural zeolite pretreated with NaCl has shown strong antibacterial activity against *E. coli*, *E. faecalis*, and coliforms [51,52].

In another study, Gördes clinoptilolite in its exchanged form was tested against two bacterial strains. Interestingly, it was observed that increasing silver content does not always result in enhanced antibacterial performance—there appears to be a threshold beyond which additional silver offers no further benefit [53]. Nevertheless, silver-exchanged clinoptilolite (Ag-clinoptilolite) stands out as a promising, cost-effective antibacterial material due to its high selectivity and efficacy, particularly against *P. aeruginosa* and *E. coli*.

Further, research on Mexican zeolite-rich tuff subjected to sodium enrichment has shown that the disinfection efficiency of silver- or copper-modified systems is influenced by both the survival kinetics of pathogens such as *E. coli* and *C. albicans*, and the release dynamics of Ag^+^ and Cu^2+^ from the zeolitic networks [54]. Similarly, Serbian natural zeolite containing 70 wt% clinoptilolite, after Na pretreatment, exhibited notable antibacterial effects against *Acinetobacter baumannii* [55].

In some cases, sodium pretreatment is combined with additional pre-functionalization steps to further enhance bactericidal action. For example, silver-clinoptilolite pretreated with oxalic acid (P1-Ag^+^) showed superior antibacterial efficacy compared to that treated with sodium hydroxide (P2-Ag^+^). This difference is attributed to variations in the degree of silver exchange and the mobility of silver ions within the zeolite matrix [56].

#### 3.1.2. Copper

Copper ion exchange in zeolitic materials can be efficiently achieved by simply immersing zeolite powders in a copper precursor solution, as demonstrated in multiple studies [18,50,51]. For example, copper-functionalized natural zeolites have shown promising antibacterial activity, particularly against *E. coli*, with enhanced performance when combined with other antimicrobial agents [57].

In one study, Iranian zeolitic tuff was treated by immersion in a copper precursor solution followed by filtration, resulting in strong antibacterial activity against two *E. coli* strains [27]. Similarly, Chilean natural zeolite underwent hydrochloric acid pretreatment prior to copper functionalization, leading to effective inhibition of *S. aureus* and *A. baumannii* [58].

Beyond ion exchange in solution, copper can also be introduced into zeolites via solid-state reactions. For instance, Georgian natural phillipsite-containing tuff from Shukhuti demonstrated complete antibacterial activity after 24 h, with significantly higher copper content compared to synthetic zeolites and natural clinoptilolite treated via traditional ion exchange [50].

As for silver, a common pretreatment to enhance copper exchange is sodium enrichment, which increases the zeolite’s ion exchange capacity. This method has been well-documented and shows broad-spectrum biocidal activity against *S. aureus*, *E. coli*, *A. baumannii*, and fungicidal activity against *C. albicans* [53,54,55,59].

To further improve copper uptake, sodium enrichment can be combined with other treatments such as plasma processing, which promotes deeper incorporation of copper into the zeolite framework and enhances antimicrobial performance [60].

Another widely used pretreatment involves chemical cleaning, which increases the surface area of natural zeolites by removing impurities from their pores and channels. Initially developed for catalytic applications [61], this method was successfully used for antibacterial purposes [62].

#### 3.1.3. Zinc and Other Metal Ions

Zinc ion exchange has also been explored to enhance the antibacterial properties of natural zeolites. In a study using Iranian zeolitic tuff, simple ion exchange with zinc resulted in measurable antibacterial activity against specific *E. coli* isolates [27]. Similarly, zinc-exchanged natural zeolite demonstrated notable antibacterial effects, particularly against *S. aureus* and *E. coli*. When further combined with silver and copper ions, the material achieved complete inhibition of *S. aureus*, *E. coli*, *P. aeruginosa*, and *C. albicans* [57].

Solid-state reaction methods have also been applied. Georgian natural phillipsite-containing tuff rock underwent a solid mixture reaction with zinc, yielding a material with strong antibacterial activity against *E. coli* [50].

In clinical research, zinc-enriched sodium-modified natural zeolite from Cuba, composed primarily of clinoptilolite and mordenite, was tested for its ability to inhibit the growth of *Helicobacter pylori*, showing promising results [63].

Turkish clinoptilolite-rich zeolite from the Gördes region was also studied. Although the material exhibited low selectivity for zinc and copper ions across most concentrations, it showed some antibacterial activity against *P. aeruginosa* following sodium enrichment and zinc exchange [53].

Another study using zeolite from the same Gördes region, pretreated with sodium enrichment and followed by zinc ion exchange, reported effective antibacterial activity against *K. pneumoniae* and *S. aureus* [64].

Additionally, Serbian natural zeolitic tuff containing clinoptilolite was modified through sodium enrichment and subsequent zinc ion exchange. After 24 h of exposure to the zinc-modified zeolite (ZnNZ), the bacterial counts of *E. coli* and *S. aureus* in treated water were reduced by six orders of magnitude [59].

Another example involving cobalt as the functional metal ion was reported by Muleja et al., in which natural zeolite was impregnated with a cobalt precursor solution followed by calcination. The resulting Co-modified zeolite exhibited a high inactivation rate against *Escherichia coli* [65].

### 3.2. Nanoparticles

Various types of nanoparticles (NPs) and their derivatives have gained significant attention for their potent antimicrobial properties. Metal-based nanoparticles such as silver (Ag), silver oxide (Ag_2_O), titanium dioxide (TiO_2_), silicon (Si), copper oxide (CuO), zinc oxide (ZnO), gold (Au), calcium oxide (CaO), and magnesium oxide (MgO) have all been shown to exhibit strong antibacterial effects in vitro, effectively inhibiting the growth of several microbial species [66].

Several antimicrobial mechanisms have been reported for nanoparticles; the primary mechanisms include: (1) disruption of the bacterial cell membrane; (2) generation of reactive oxygen species (ROS); (3) penetration of the bacterial membrane; and (4) intracellular interactions with critical biomolecules such as DNA and proteins [67,68]. Furthermore, certain semiconductor nanoparticles, such as TiO_2_ and ZnO, can exhibit photocatalytic antimicrobial activity under suitable irradiation conditions, promoting the generation of reactive oxygen species (ROS) that induce oxidative damage to bacterial membranes, proteins, and other cellular components [69].

For nanoparticles to exert their antimicrobial effects, direct contact with bacterial cells is essential. This interaction may occur via electrostatic attraction, van der Waals forces, receptor–ligand binding, or hydrophobic interactions. Once in contact, NPs can cross the bacterial membrane and interfere with internal cellular structures, ultimately inducing oxidative stress, altering membrane permeability, disrupting electrolyte balance, inhibiting enzyme activity, and modifying gene expression [67]. In dissolution-driven mechanisms, nanoparticles act as carriers for metal ions, facilitating their localized delivery. Upon adhesion to biological surfaces, they can release elevated concentrations of metal ions at the interface or enable a sustained and controlled release over time, thereby enhancing their activity [70]. It is important to note that bacterial cells, typically exhibiting micrometer-scale dimensions [71], cannot penetrate the angstrom-scale micropores of zeolites. Therefore, direct contact-mediated antimicrobial activity is primarily associated with active species exposed on the external zeolite surface, whereas metal ions located within the internal pore structure can contribute to microbial inactivation mainly through their release and subsequent diffusion into the surrounding environment.

Additionally, physicochemical characteristics—such as particle size and surface charge—play a vital role. Smaller nanoparticles generally display stronger bactericidal effects, and positively charged NPs tend to adhere more readily to negatively charged bacterial surfaces, enhancing their antimicrobial action [66].

Zeolites, due to their rigid, three-dimensional porous frameworks, provide an ideal support matrix for the uniform dispersion of nanoparticles. This structural characteristic allows for atomic-level distribution of metal NPs while maintaining high performance [72]. Furthermore, the nanoscale cavities and channels within zeolites restrict nanoparticle growth, helping stabilize their size. Under appropriate conditions, metal cations can migrate through the zeolite structure and precipitate on the external surface as metal or metal oxide nanoparticles [73].

Natural zeolites impregnated with silver nanoparticles (Ag NPs) have found applications in several fields, including water purification, agriculture, and antimicrobial coatings and paints [74].

#### 3.2.1. Silver Nanoparticles

Silver nanoparticles (AgNPs) can be effectively supported on natural zeolites using a two-step strategy: ion exchange of Ag^+^ ions followed by their reduction through thermal or chemical means. This approach has been widely applied across various natural zeolites, particularly clinoptilolite and chabazite, demonstrating potent antibacterial performance.

For example, clinoptilolite-rich tuff from Mexico was first enriched with sodium, then exchanged with silver ions, and subsequently reduced at either 550 °C or 700 °C under a hydrogen atmosphere (5 mL/min) for 4 h. The resulting metallic AgNP-zeolite composite showed excellent biocidal activity against *E. coli*, reducing bacterial count by 2 log_10_ within 60 min at a concentration of 1.7 mg/mL (with an initial concentration of ~200 CFU/mL). A higher concentration (6.7 mg/mL) was required to achieve complete inhibition of *Salmonella* [75].

In another study, natural zeolitic rock from the East Rhodopes region in Bulgaria was ion-exchanged with silver ions and annealed in air at 200, 400, and 600 °C for 2 h. The process yielded AgNPs of two distinct size ranges—small particles (3–5 nm) embedded within microstructural defects and larger particles (20–25 nm) located on the surface. These composites were effective at inhibiting *E. coli* growth even in low concentrations in peptone water [76].

Natural chabazite from Mexico underwent silver ion exchange via stirring in a 0.01 M AgNO_3_ solution at room temperature, followed by thorough washing and drying at 50 °C for 5 h. Thermal annealing at 400 °C in air for 1 h produced silver-supported chabazite. The resulting composite exhibited potent antibacterial activity against multiple pathogenic bacteria, including *S. typhimurium* and *S. flexneri* [73].

In another approach, silver ions were introduced into natural clinoptilolite by ion exchange and wet impregnation, followed by chemical reduction using sodium borohydride (NaBH_4_). The final material demonstrated antimicrobial activity specifically against *E. coli* [77].

Green synthesis routes have also been employed to reduce the environmental impact of AgNP functionalization. For instance, AgNPs were synthesized using *Moringa oleifera* seed extract and subsequently immobilized onto natural zeolite by immersing the zeolite in the AgNP suspension under stirring. The resulting AgNP-zeolite (NZ-AgNPs) composite required four times less silver to exhibit the same bactericidal effect against *E. coli* compared to free AgNPs [74,78].

Another green route involved synthesizing clinoptilolite–AgNP composites in situ using *Vaccinium macrocarpon* (cranberry) fruit extract as the reducing agent, resulting in stable AgNP formation directly in the presence of zeolite under stirring [79].

Additionally, natural clinoptilolite has been functionalized with silver nanoparticles via in situ reduction using tannic acid. This method successfully produced an antibacterial material effective against both *S. epidermidis* and *E. coli*, using a simple, low-temperature and sustainable approach [26].

#### 3.2.2. Copper Oxide

Copper oxide nanoparticles (CuO NPs) can be supported on natural zeolites using various functionalization strategies, including direct impregnation and ion exchange followed by thermal treatment. These methods yield zeolite-based composites with demonstrated antibacterial activity.

In one approach, pre-synthesized CuO nanoparticles (20–100 nm in size) were mechanically agitated with natural zeolite to achieve surface impregnation. This simple coating technique resulted in a CuO–zeolite composite with effective antibacterial activity against *Staphylococcus aureus* and *Acinetobacter baumannii* [58].

A second method was employed on natural Indonesian zeolite, which was first soaked in a 0.5 M CuSO_4_·5H_2_O solution for two days, followed by drying at 60 °C overnight. Half of the samples were then subjected to heat treatment at approximately 500 °C for 2 h, leading to the formation of a CuO and Cu_2_O/zeolite composite. This material demonstrated strong antibacterial performance without any degradation in quality [80].

In a third study, natural zeolitized tuff from the Zlatokop sedimentary deposit in Serbia—composed primarily of clinoptilolite with plagioclase feldspar and quartz impurities—was first enriched with sodium using a NaCl solution. This Na-clinoptilolite was then treated with an aqueous CuCl_2_ solution to load the metal ions. The metal-loaded zeolite was subsequently dehydrated at 550 °C in air for 1 h, resulting in the formation of Cu_2_O on the zeolite surface. The final material exhibited notable antibacterial activity and demonstrated the feasibility of incorporating other metal oxides such as NiO and ZnO using the same procedure [81].

#### 3.2.3. Other Nanoparticles

Nickel oxide and zinc oxide nanoparticles have also been incorporated into natural zeolites using the same multi-step procedure previously applied for copper and is reported in the same paper. The raw material was a natural zeolitized tuff from the Zlatokop sedimentary deposit in Serbia, containing approximately 70 wt% clinoptilolite. The zeolite was first enriched with sodium via treatment with a NaCl solution to enhance its cation exchange capacity. Subsequently, it was treated with aqueous solutions of metal chlorides to produce metal-loaded zeolites. These were then dehydrated in air at 550 °C for 1 h, resulting in the formation of ZnO, or NiO nanoparticles. The final composites exhibited antimicrobial activity, particularly against *Escherichia coli* [81].

Gold nanoparticles have also been successfully supported on natural zeolites. In one study, a clinoptilolite-rich tuff from Etla, Oaxaca (southeastern Mexico) was first converted into its protonated form (H-zeolite). The H-zeolite was then suspended in a commercially obtained colloidal gold solution containing 5 nm Au nanoparticles. The suspension was stirred for 90 min, after which the solid was separated by centrifugation, washed with distilled water, dried at 50 °C, calcined for 4 h, and finally reduced at 500 °C under a hydrogen flow. The resulting Au-zeolite composite demonstrated excellent biocidal activity, effectively eliminating *Escherichia coli* and *Salmonella typhi* in short contact times [82].

A Romanian zeolitic mineral from Mirsid was used as a support for TiO_2_-based functionalization. Initially, undoped TiO_2_ nanocrystals were synthesized via a fast-hydrothermal method. These nanocrystals were subsequently incorporated into natural zeolite (in its sodium-enriched form) through a solid-state reaction under fast-hydrothermal conditions. The TiO_2_ particles were found to be distributed both as agglomerated clusters on the surface and within the channels of clinoptilolite. Evaluation of the antibacterial performance against *Enterococcus faecalis* revealed that the bactericidal effect was dependent on both the dose of the material and the duration of irradiation [83].

### 3.3. Antibacterial Molecules

Nitric oxide has demonstrated broad-spectrum antimicrobial activity against a wide variety of pathogens, including bacteria, viruses, fungi, and parasites [84]. A zeolitic tuff sourced from Gördes (Western Anatolia) was functionalized with NO by exposing the natural zeolite to a continuous flow of NO gas (20 mL/min, 99.5% purity) at 30 °C. The NO-loaded zeolite exhibited antibacterial activity against both *Escherichia coli* and *Bacillus subtilis* [85].

The antimicrobial activity of benzalkonium chloride, a quaternary ammonium compound, is well established [86]. In one study, natural zeolite containing 70 wt% clinoptilolite from the Zlatokop sedimentary deposit in Serbia was used. The material was first enriched with sodium to enhance the cation exchange capacity of clinoptilolite. The sodium-enriched zeolite (NaNZ) was then treated with an aqueous solution of benzalkonium chloride. The modified material was separated by filtration and dried overnight. The resulting BKC-zeolite composite demonstrated antibacterial activity against *Acinetobacter baumannii* [55].

Focusing on natural compounds, polyphenols have proven to be promising alternatives to harsh chemical agents for antibacterial applications [87]. By functionalizing clinoptilolite through supercritical solvent impregnation with thymol or carvacrol, the natural zeolitic tuff acquired antibacterial properties effective against both Gram-positive and Gram-negative bacteria, highlighting its potential as a water disinfectant [88].

### 3.4. Combined Approaches

The combination of multiple antimicrobial agents offers several advantages, including enhanced biological activity, reduced toxicity, and greater antimicrobial effectiveness due to the complementary mechanisms of action. In synergistic interactions, one agent enhances the activity of another, resulting in more efficient and potent antimicrobial effects [89].

In the study by Torres-Giner et al., previously noted for ion exchange of silver, copper, and zinc on natural zeolite, a multimetallic strategy was also evaluated. The ternary Ag–Cu–Zn-zeolite composite demonstrated superior antimicrobial efficacy, achieving complete microbial inhibition with a reduced amount of silver, highlighting the synergistic benefits of the multimetal approach [57].

Similarly, in the previously mentioned work involving TiO_2_-zeolite nanocomposites, silver doping was also introduced. The Ag–TiO_2_–zeolite composite achieved the highest antimicrobial performance, with sterile culture plates obtained after 180 min of irradiation, confirming the enhanced activity through combined functionalities [83].

These reported studies are summarized in Table 1.

A graphical overview of the functionalization of natural zeolites with ions, nanoparticles, and antibacterial molecules is presented in Figure 1.

Although a wide range of functionalization strategies has been explored, their translation from laboratory-scale studies to real-world applications remains a critical challenge. Among antimicrobial metal ions, silver is widely recognized as one of the most effective [90]. However, its extensive use has raised concerns regarding the development of bacterial resistance [91], as well as regulatory limitations on its concentration in water due to potential environmental and health risks [92]. In comparison, copper (Cu^2+^) and zinc (Zn^2+^) ions have been less extensively studied as antimicrobial agents in this context and are generally associated with different regulatory thresholds in drinking water, often reflecting a more favorable toxicity profile [93,94]. From a practical perspective, the selection of antimicrobial metal ions should also consider the balance between effectiveness, cost, and long-term stability. While silver generally exhibits higher antimicrobial activity, copper and zinc may offer more economically accessible alternatives [95], with the suitability of each system depending on the intended application and the stability of the incorporated metal species.

In this context, nanoparticles represent a valuable alternative or complementary strategy. In aqueous environments, they can act as reservoirs for the gradual release of metal ions, thereby avoiding rapid burst release and improving long-term effectiveness [96]. In addition, metallic and metal oxide nanoparticles can exert antimicrobial activity through direct contact with bacterial cells [67], making them effective even in conditions where ionic release is limited, such as dry environments. Furthermore, inorganic nanoparticles may exhibit catalytic properties, enabling simultaneous degradation of chemical pollutants and microbial inactivation [97,98].

Moreover, resistance phenomena have also been reported for nanoparticle-based systems [99]. Although silver nanoparticles remain among the most widely used and effective antimicrobial agents [100], these limitations highlight the importance of adopting combined or multi-functional strategies. Such approaches can enhance antimicrobial performance while reducing the required metal concentration, thereby facilitating compliance with regulatory limits, particularly in water treatment applications.

Health risks associated with the potential leaching of nanoparticles remain a significant concern and pose challenges for regulatory frameworks [101]. In addition, standardized testing methods specifically designed to assess nanoparticle release from nano-enabled materials are still lacking [102]. In this context, functionalization strategies that go beyond simple impregnation—such as approaches that promote stronger binding or encapsulation of active species—may be preferable to minimize nanoparticle release and improve the environmental and operational safety of these systems. A schematic representation of the different mechanisms and limitations is reported in Figure 2.

Similarly, antimicrobial molecules present additional limitations that may hinder their practical application. For instance, natural compounds such as polyphenols often exhibit limited chemical stability [103], while widely used biocidal agents such as benzalkonium chloride have been associated with cytotoxic effects on human cells [104]. Nitric oxide, although effective as an antimicrobial agent, also raises concerns due to its intrinsic toxicity [105].

## 4. Applications in Air Decontamination

### 4.1. Air Decontamination

Air quality deterioration and airborne infectious agents are critical public health concerns. Key chemical pollutants commonly found in the air include ozone (O_3_), nitrogen oxides (NO_x_), sulfur oxides (SO_x_), volatile organic compounds (VOCs), and particulate matter (PM) [106].

Volatile organic compounds (VOCs) represent a large group of gaseous organic trace substances released from both natural and human-related sources, and they can also form through chemical processes in the atmosphere. VOCs are among the most extensively studied indoor air contaminants [107].

The World Health Organization (WHO) has categorized VOCs into four classes based on boiling point ranges: very volatile organic compounds (VVOCs, <0 to 50–100 °C), volatile organic compounds (VOCs, 50–100 to 240–260 °C), semi-volatile organic compounds (SVOCs, 240–260 to 380–400 °C), and particulate organic matter (POM, >380 °C) [108].

VOCs are known for their high diffusivity, toxicity, and potential carcinogenicity, posing risks to ecosystems and human health [109,110]. They are major contributors to ground-level ozone and secondary organic aerosol (SOA) formation. VOCs can be either biogenic (emitted by plants and microbes) or anthropogenic (originating from industrial activities, fossil fuel combustion, and solvents) [111].

Humans are exposed to VOCs via inhalation, ingestion of polluted food or water, and dermal contact with contaminated soils or waste emissions [112,113]. As a result, emission control and purification are vital. Techniques such as biofiltration, adsorption, membrane separation, and catalytic oxidation (including photocatalysis, ozonation, microwave-assisted processes, plasma-based degradation, and hybrid catalytic systems) are employed. Supported metal catalysts are commonly used, with active phases including noble, transition, or rare earth metals, and supports such as TiO_2_, SiO_2_, Al_2_O_3_, CeO_2_, activated carbon, metal–organic frameworks (MOFs), and zeolites [114].

Alongside VOCs, particulate matter (PM) presents another major threat to air quality and human health. Particulate matter, in fact, especially fine particles, can evade the respiratory system’s defense mechanisms and accumulate in the lungs [115]. PM is notorious for adsorbing toxic heavy metals and increasing the incidence of respiratory and cardiovascular diseases, as well as cancers [116]. Filtration of PM is achieved through mechanisms like sieving, diffusion, gravitational settling, inertial impaction, interception, and electrostatic attraction [117]. Filters made from materials such as activated carbon, woven fabrics, and polymers are widely used due to their flexibility, cost-efficiency, and adaptability. Fibrous and porous filters continue to evolve, although advancements are still needed in transparency, reusability, and reducing pressure drop. Recently, innovative porous materials like MOFs and zeolites have shown promising capabilities for PM removal.

Airborne pathogens also pose serious health threats. Viruses, bacteria, and fungi can become aerosolized and spread through droplets or airborne transmission pathways [118]. Droplet transmission involves large respiratory droplets that quickly settle on surfaces, whereas airborne transmission involves smaller particles that can remain suspended for extended periods and travel longer distances. Numerous studies have confirmed that respiratory infections—including measles, influenza, RSV, rhinovirus, adenovirus, enterovirus, SARS-CoV, MERS-CoV, and SARS-CoV-2—can be transmitted via aerosols [119].

Crucially, air pollution can exacerbate the spread of these pathogens. Airborne microorganisms can adhere to particulate matter (PM), which acts as a carrier that prolongs their suspension in the air and facilitates wider dispersion. This interaction between bioaerosols and pollutants amplifies the risk of infection [120].

Indoor environments with poor ventilation significantly increase the risk of exposure to airborne pathogens. Heating, ventilation, and air conditioning (HVAC) systems, while designed to improve air circulation, can inadvertently contribute to the dispersion and prolonged suspension of infectious aerosols following respiratory events such as coughing [121]. As such, HVAC systems are critical engineering tools for controlling indoor bioaerosol concentrations [122]. However, if not properly maintained, air filters within these systems can accumulate microorganisms, creating a favorable environment for bacterial growth and potentially becoming secondary sources of infection [123].

A wide range of air purification strategies have been developed for microbial decontamination, each presenting specific advantages and limitations. Filtration remains one of the most common approaches, using materials like activated carbon and polypropylene fibers to physically trap airborne microorganisms; however, it does not inactivate the captured microbes and requires frequent maintenance and replacement [124]. Thermal disinfection, involving moist or dry heat, offers effective microbial inactivation without producing harmful byproducts, although it is highly energy-intensive [125]. Other methods, such as ultraviolet (UV) irradiation and plasma-based technologies, have also shown strong antimicrobial performance but may lead to the formation of unwanted gaseous byproducts and secondary particles [125,126]. Microwave irradiation can damage microbial cell structures through localized heating, although its effectiveness in air environments is limited without the use of microwave-absorbing materials [127]. While ozone treatment and chemical aerosolization are effective disinfectants, their use raises safety concerns due to the potential toxicity of the reactive species they release [118].

To overcome the limitations of these conventional approaches, nanomaterials are being investigated for their enhanced antimicrobial capabilities. Nonetheless, their potential cytotoxicity and environmental risks following release remain significant concerns [125].

### 4.2. Use of Zeolites in Air Filtration

Zeolites, owing to their high thermal and hydrothermal stability, large ion-exchange capacity, tunable acidity, porosity, and high surface area, are promising for catalytic and adsorption-based air purification, particularly in VOC oxidation [114]. Modifications, such as copper exchange in clinoptilolite and mordenite [128], ceria promotion in Mn/clinoptilolite catalysts [129], and Pd/C–clinoptilolite–CeO_2_ formulations [130], have significantly improved VOC removal, with some systems achieving up to 98% abatement and long-term stability. Natural Chilean zeolite mixtures [131] showed reduced efficiency under humidity, but pretreatment restored performance. Metal oxide loading, particularly MnO_x_ [132], enhanced toluene combustion activity, while integration of zeolite granules in biofilters [133] improved acetone pre-removal. Surface modifications via ion exchange and photocatalytic functionalization [134] further boosted VOC decomposition, enabling applications such as indoor air purification textiles.

Beyond VOC control, zeolites have also shown strong potential for particulate matter (PM) removal, rivaling metal–organic frameworks (MOFs) [135,136,137]. FAU-type NaX and NaY zeolites coated on fabrics [138] effectively captured PM_2.5_ and PM_10_ through electrostatic interactions, with NaX outperforming other formulations. Potassium-exchanged synthetic zeolites on cotton [117] demonstrated high PM removal efficiency and biodegradability, supporting the development of sustainable, scalable air filtration technologies using both natural and synthetic zeolites.

In commercial buildings, indoor air quality is primarily managed through HVAC systems, where mechanical filtration is widely used to remove particulate matter (PM) with relatively low energy consumption. These systems are generally effective for particle removal; however, they are less efficient in addressing gaseous pollutants, particularly volatile organic compounds (VOCs), which often require additional treatment strategies [139].

To overcome these limitations, adsorption-based materials such as activated carbon are commonly integrated into air purification systems. Activated carbon offers high surface area and adsorption capacity, but it exhibits a broad pore size distribution, limited selectivity toward specific pollutants, and lacks ion-exchange capability. Moreover, once saturation is reached, previously adsorbed compounds may be released back into the environment, requiring frequent replacement and reducing long-term operational stability [140].

In this context, zeolite-based materials represent a promising alternative or complementary solution due to their well-defined pore structure, ion-exchange capacity, and potential for functionalization with antimicrobial agents. These features enable not only the adsorption of pollutants but also their transformation or inactivation, which is particularly relevant for bioaerosol control. Recent studies have demonstrated the feasibility of integrating zeolites into engineered filtration systems, highlighting their potential for incorporation into HVAC units and hybrid filter configurations [140,141].

#### Microbial Decontamination

In addition to their role in particulate matter removal, antimicrobial zeolites have gained increasing attention for their ability to suppress microbial growth and limit the transmission of airborne pathogens in air filtration systems. When functionalized with antimicrobial metal ions such as silver (Ag^+^), copper (Cu^2+^), and zinc (Zn^2+^), zeolites exhibit enhanced antimicrobial properties that have been validated across various studies.

One of the earliest investigations into the use of antimicrobial zeolites in air filters was conducted by Kim et al. (2012) [142], who developed a platinum-zeolite-coated filter using nonwoven polypropylene and rayon materials. Their tests, which included both traditional inoculation and bioaerosol assays, demonstrated rapid inactivation of the influenza virus, though *Bacillus subtilis* spores were more resistant. Notably, microbial removal occurred only through direct contact with the functionalized filter surface.

The integration of zeolite-based antimicrobial surfaces into HVAC systems has also been explored. Rizzetto et al. [143] evaluated Ag^+^-zeolite-coated polyurethane panels and observed a marked decrease in airborne bacterial load within treated ducts compared to conventional zinc-galvanized systems. Air exiting the Ag^+^-zeolite-lined ducts contained five times fewer bacteria than air from untreated systems. Similarly, Tinteri et al. (2012) [144] confirmed the long-term bactericidal activity of silver-zeolite coatings, which remained effective for over 2.5 to 3 years.

Zendehdel et al. [145] developed a nanofiber-based antimicrobial air filter by incorporating metal-zeolite (NZY) composites into electrospun PVDF-PVP fibers. The zeolite matrix served as a robust host, limiting metal ion leaching while preserving antimicrobial activity. Among the different formulations tested, the trimetallic system (Zn-Cu-Ag) demonstrated the highest antibacterial performance against *S. aureus* and *E. coli*, suggesting synergistic interactions between the three metals.

A recent innovation by Fuentes-Alburquenque et al. (2024) [146] involved a copper-zeolite antimicrobial coating applied in detention cells. Over five months, a significant reduction in airborne coliform bacteria was recorded, highlighting its practical application in air sanitization within high-risk environments.

Silver-functionalized zeolites (AgZ) have also shown efficacy in both VOC removal and microbial control. Shen et al. [147] assessed AgZ filters in a medical setting and reported effective elimination of VOCs, bacteria, and fungi after 120 h. They proposed AgZ filters as a feasible strategy for infection control in hospital air systems. Earlier efforts by Cheng et al. and Wang et al. [148,149] also demonstrated the antimicrobial potential of AgZ in indoor environments.

Shirmohammadi-Bahadoran et al. (2018) [150] presented a novel approach involving the dry electro-deposition of clinoptilolite/AgNPs onto 3D polyester spacer fabric for air filtration. This method, free from solvents, binders, and chemical additives, allowed for simultaneous removal of SO_2_ and inactivation of airborne *P. aeruginosa* and *S. aureus*, while preserving the functional integrity of the nanosilver–zeolite composite.

A notable example of viral inactivation is the work by Kim et al. (2022) [151], who developed a TiO_2_/H-ZSM-5 zeolite composite bead filter activated by UVA-LEDs. Their photocatalytic air purifier successfully removed indoor VOCs and inactivated viruses, including SARS-CoV-2, highlighting the potential of zeolite-based photocatalytic materials in tackling both chemical and biological indoor air pollutants.

Although the studies presented in this section pertain exclusively to synthetic zeolites, previous results have demonstrated that natural zeolites are also effective in removing VOCs and PM and can be functionalized with antimicrobial agents. These findings suggest that a combined approach for air purification is equally applicable to natural zeolites.

This multifunctional potential arises from the ability of the same zeolitic matrix to contribute to different purification processes through distinct mechanisms. VOC adsorption primarily occurs within the accessible microporous structure, where small molecules can diffuse and interact with internal adsorption sites [152]. In contrast, particulate matter is captured through physical filtration mechanisms associated with the external surfaces and the macroscopic structure of the filtering system [153]. Microbial inactivation, on the other hand, is mainly associated with interactions between microorganisms and antimicrobial species exposed on the external zeolite surface, as well as the release of bioactive metal ions [71]. Indeed, due to their micrometer-scale dimensions, bacterial cells cannot penetrate the angstrom-scale micropores of zeolites. Furthermore, the incorporation of metal or metal oxide nanoparticles can introduce additional reactive sites, including on the external surface, potentially promoting catalytic or photocatalytic degradation of organic contaminants under suitable operating conditions [154]. Therefore, the same zeolitic matrix can support complementary purification mechanisms, although the effectiveness of each process must be independently demonstrated.

Nevertheless, despite these promising results, studies investigating the long-term antimicrobial stability of zeolite-based materials remain limited. Prolonged operation may lead to the depletion of active antimicrobial species, pore blockage, and surface fouling, potentially compromising their effectiveness over time. Therefore, further investigations under realistic operating conditions, including repeated use and regeneration cycles, are needed to assess antimicrobial durability, material stability, and potential metal release. The incorporation of zeolites should also account for potential increases in pressure drop which can be influenced by the structural and surface characteristics of porous filters, as these factors may influence long-term filtration performance and material durability [155].

## 5. Applications in Water Decontamination

### 5.1. Water Decontamination

Water contamination represents a pressing global challenge, stemming from both natural and anthropogenic (human-induced) sources [156]. From a chemical perspective, water pollutants are generally categorized into four main groups:

- Inorganic contaminants (e.g., heavy metals);
- Organic contaminants (e.g., pesticides, volatile organic compounds);
- Radiological contaminants;
- Biological contaminants (e.g., bacteria and protozoa) [157].

The occurrence and concentration of naturally derived contaminants in groundwater are closely linked to the geological substrates through which water flows, as well as the quality of the recharge sources. Common naturally occurring pollutants include magnesium, calcium, chloride, arsenate, fluoride, nitrate, and iron. Their effects on water quality are determined by their specific chemical characteristics and concentrations [158].

Beyond natural sources, anthropogenic activities—particularly those related to industry and agriculture—introduce a wide array of hazardous substances into aquatic systems. These include heavy metals, synthetic dyes, insecticides, fertilizers, and various chemical compounds. Moreover, the improper handling, storage, and disposal of household products such as paints, detergents, solvents, oils, pharmaceuticals, disinfectants, pool chemicals, pesticides, batteries, and fuels further contribute to the contamination of groundwater [159].

Among these, heavy metals pose a serious threat to human health. Once ingested through polluted water or contaminated food, these metals can bind to organic molecules in the body, forming toxic compounds that interfere with cellular functions and cause long-term damage [160].

Industrial processes also release persistent organic pollutants (POPs)—organic compounds that resist biodegradation and accumulate in ecosystems. Due to their toxic effects and environmental persistence, POPs have recently gained public and scientific attention, especially because many of them act as endocrine-disrupting chemicals, impairing reproductive and hormonal functions in both humans and wildlife [161].

Microbial contamination remains another critical concern. Pathogens such as bacteria, viruses, and protozoa frequently infiltrate water supplies through human and animal waste, often without immediate detection [157]. A lack of access to safe drinking water and adequate sanitation is a major driver of numerous preventable diseases, including diarrhea, legionellosis, hepatitis, malaria, and dengue [162]. According to the World Health Organization (WHO), waterborne diarrheal illnesses alone are responsible for nearly one million deaths annually [163]. In addition to their human cost, water-related diseases impose substantial economic burdens on affected communities. This global crisis is further exacerbated by population growth, climate change, deforestation, ecosystem degradation, and microbial evolution—trends that are expected to worsen the situation in the coming decades [164].

To address microbial contamination, conventional water disinfection methods are widely employed. These include chemical treatments using chlorine, chlorine dioxide, ozone, and peracetic acid, as well as physical techniques such as ultraviolet (UV) radiation. While effective, these approaches can generate disinfection by-products that may pose additional health and environmental risks [165].

As a result, there is growing interest in chemical-free alternatives for water treatment. Innovative approaches include the use of natural essential oils with antimicrobial properties [166], solar disinfection methods [167], and natural filtration materials such as sand, clay, and zeolites [168,169,170].

Among these materials, natural zeolites have shown particularly strong potential due to their unique cation-exchange properties. These microporous aluminosilicate minerals can effectively remove dissolved ions from water by exchanging them with cations present on their surfaces. Furthermore, the ion-exchange performance of natural zeolites can be significantly enhanced through modification techniques, such as sodium enrichment or surface treatment, broadening their application in water purification processes [171].

### 5.2. Use of Zeolites in Water Purification

Natural zeolites possess notable ion-exchange capacity, enabling the adsorption of heavy metals such as Pb^2+^, Cu^2+^, and Cd^2+^ from aqueous environments [172], with studies confirming their effectiveness in industrial wastewater treatment and reusability after NaCl regeneration [173]. Clinoptilolite, one of the most abundant types, efficiently removes Cd, Fe, and Ni [174], while modifications such as acid treatment, thermal activation, and surfactant or metal reagent functionalization enhance adsorption capacity [175], thermally treated samples achieving up to 98% Cu removal with residual concentrations below drinking water limits [176]. Beyond metals, zeolites effectively remove ammonium from greywater and swimming pool wastewater [177], as demonstrated by Iranian [178] and Turkish clinoptilolite studies [179]. For organic pollutants, unmodified zeolites have low affinity due to their hydrophilic, negatively charged surfaces, but cationic surfactant functionalization significantly improves adsorption [172,180]; for example, SDBAC-modified clinoptilolite achieved 98% removal efficiency [181]. Zeolites such as mordenite, erionite, chabazite, and clinoptilolite are also used for radionuclide remediation, particularly for cesium isotopes like ^137^Cs and ^135^Cs in radioactive waste [182], leveraging their high sorption capacity, thermal and mechanical stability, and low cost—attributes that have been applied in nuclear accident remediation [183]. Functionalized clinoptilolite further enhances ^137^Cs uptake, reinforcing its applicability in radioactive waste treatment [184].

The most extensive real-world applications of natural zeolites have been reported, indeed, in the treatment of radioactive wastewater, particularly following the Chernobyl and Fukushima nuclear disasters [185]. In these contexts, numerous pilot- and near-industrial-scale studies, starting from the 1980s, have demonstrated the feasibility of using zeolites as effective sorbents for radionuclide removal [186,187]. These examples highlight the potential of natural zeolites for large-scale environmental applications, although their use for antimicrobial decontamination has not yet reached comparable levels of implementation.

In addition to packed-bed or column-based systems, zeolites have also been explored as components of membrane technologies. Their application in this field can be broadly classified into self-supported zeolite membranes, inorganic-supported zeolite membranes, and mixed-matrix membranes in which zeolite crystals are dispersed within a polymeric matrix. However, self-supported zeolite membranes are typically fabricated on removable supports such as cellulose or Teflon and tend to exhibit poor mechanical strength, making them unsuitable for most industrial applications [188].

In particular, the performance of natural zeolites in real wastewater systems may differ significantly from laboratory conditions due to the presence of competing ions, and fluctuating operating parameters [189]. Furthermore, the negatively charged aluminosilicate framework generally favors cation exchange, while limiting the adsorption of certain anionic contaminants. Although surface modifications can improve adsorption selectivity, additional processing steps may increase treatment costs [190]. The presence of organic matter may also interfere with adsorption and ion-exchange processes by occupying accessible sites or interacting with released metal ions. In particular, complexation with inorganic species or dissolved organic matter may reduce the fraction of released metal ions that remain freely available to exert antimicrobial activity [191].

Flow conditions may also influence contaminant removal by affecting contact time and mass transfer, highlighting the importance of evaluating zeolite performance under dynamic operating conditions [192].

Moreover, regeneration efficiency and the associated chemical and economic requirements represent important factors to consider for the long-term sustainability of zeolite-based wastewater treatment systems [190].

#### Microbial Decontamination

Silver modified zeolites

Among the various antimicrobial agents explored for water disinfection, silver (Ag) has emerged as one of the most effective, particularly due to its strong bactericidal properties. When incorporated into natural zeolite frameworks, silver not only benefits from the structural and ion-exchange characteristics of the host material but also gains a stable and active platform for microbial inactivation. Research in recent years has consistently highlighted the promising performance of Ag-modified natural zeolites in targeting common waterborne pathogens.

For example, Aparicio-Vázquez et al. synthesized silver-enriched zeolite-rich tuffs under different experimental conditions and confirmed their strong antimicrobial activity against *E. coli* [193]. Supporting this, Syngouna et al. demonstrated the broad-spectrum potential of Ag-zeolites, reporting significant antibacterial effects against both *E. coli* and *E. faecalis*, two widely recognized indicators of fecal contamination [194]. In a comparative study, Ivanković et al. evaluated silver- and copper-exchanged clinoptilolite zeolites and found that silver-treated variants were markedly more effective in eliminating *Acinetobacter baumannii*, a pathogen commonly associated with hospital-acquired infections [195].

Expanding on this body of research, Đolić et al. assessed a range of silver-activated sorbents, including zeolite, activated carbon, and TiO_2_. Their results showed that Ag-zeolite composites outperformed all others in neutralizing *Staphylococcus aureus* and *E. coli*. However, the removal of yeasts such as *Candida albicans* remained limited across all tested materials [196].

While these findings confirm the strong antimicrobial potential of Ag-zeolites, more recent studies have shifted attention to the role of water chemistry in modulating their performance. For instance, Al-Rawajfeh et al. demonstrated that silver nanoparticles (AgNPs) could be effectively integrated into natural zeolites, producing materials capable of both bromide removal and microbial disinfection—a dual functionality particularly relevant in complex water matrices [197]. Building on this, Pérez-Jiménez et al. examined the effect of chloride ions on antimicrobial efficacy and found that their presence could significantly delay the bactericidal action of AgNPs against *Escherichia coli* [198].

Further emphasizing the importance of water composition, Albarrán et al. investigated how electrical conductivity influences the antimicrobial activity of silver-modified natural zeolites. Their results indicated that the efficacy of each Ag-zeolite composite is closely related to its physicochemical characteristics, which govern its response under different conductivity conditions [199].

Taken together, these studies highlight not only the versatility and high efficacy of Ag-modified natural zeolites but also the critical influence of water chemistry in determining their real-world antimicrobial performance, reinforcing their value as a promising material for advanced and sustainable water treatment technologies.

Copper-modified zeolites

In addition to silver, copper-exchanged zeolites have shown considerable potential as antimicrobial agents in water treatment applications. Several studies have investigated their performance under various environmental and operational conditions. Chandrasena et al. (2017) evaluated Cu-doped zeolite filters within stormwater biofiltration systems and found that their disinfection efficiency was influenced by factors such as vegetation type and microbial interactions [200]. Similarly, Blel et al. demonstrated the bactericidal and antifouling properties of Cu-mordenite-coated alumina supports, which were effective in both static and dynamic experimental conditions [201]. In a large-scale field study, Li et al. reported that biofilters amended with copper-zeolite exhibited a 53% increase in *E. coli* removal, particularly under intermittent dry weather conditions, highlighting the robustness of Cu-zeolite systems in fluctuating flow environments [202]. Furthermore, Hrenović et al. compared the antimicrobial efficacy of Cu-, Zn-, and Ni-modified zeolite tuffs, finding that Cu and Zn offered effective antibacterial activity, whereas Ni-modified zeolites performed poorly due to excessive ion leaching, which limited their practical use [59]. These findings collectively support the application of Cu-exchanged zeolites as promising antimicrobial materials in decentralized and nature-based water treatment systems.

Multimetal solutions and metal comparisons

The antimicrobial performance of natural zeolites doped with metal nanoparticles has been extensively explored, particularly with silver (Ag), copper (Cu), and zinc (Zn). Martínez-Vieyra et al. synthesized Ag-, Cu-, and Zn-loaded natural zeolites using gamma irradiation, and found that Ag-doped zeolites exhibited the highest antimicrobial activity, effectively inhibiting *Salmonella typhimurium* and *Candida albicans* [203]. In a comparative study, Milenković et al.evaluated both natural and synthetic zeolites doped with the same metals. Their results confirmed that silver was consistently the most effective antimicrobial agent, regardless of the zeolite type. The study also reported minimal metal ion leaching, indicating that the observed antimicrobial effects were likely due to surface-bound metal species rather than dissolved ions [27]. These findings reinforce the role of metal-functionalized zeolites, particularly silver-based systems, as effective materials for pathogen control in water treatment applications.

Other antimicrobial agents

In recent years, a variety of metal-doped and modified natural zeolites have been explored for their antimicrobial and water treatment capabilities. Amariei et al. (2022) developed a ZnO-loaded fly ash-based zeolite coating on glass substrates, which exhibited effective photocatalytic and antimicrobial activity against *Escherichia coli* and *Staphylococcus aureus* [204]. Meanwhile, Kahol et al. evaluated a biofiltration system composed of water hyacinth, gravel, and natural zeolite; however, the system proved ineffective in reducing coliform bacteria levels [205]. In a separate approach, Li et al. modified natural zeolite using wood vinegar, reporting enhanced heavy metal removal alongside improved antimicrobial performance [206]. Yu et al. (2018) incorporated D-tyrosine-loaded FAU-type zeolite nanoparticles into membrane materials, successfully inhibiting biofilm formation on membrane surfaces, a key issue in membrane fouling [207]. Additionally, Lima et al. supported gold nanoparticles on various zeolite types, including clinoptilolite, mordenite, and faujasite, and observed strong antibacterial activity against *E. coli* and *Salmonella typhi*, with the zeolite support structure playing a significant role in determining the level of antimicrobial effectiveness [82]. These studies highlight the versatility and tunability of natural zeolites as platforms for advanced water treatment applications, particularly when functionalized with antimicrobial agents.

Regarding real-world applications, the use of natural zeolites is subject to regulatory frameworks that govern their deployment in wastewater technologies [208]. To date, their most extensive large-scale application in wastewater treatment has been observed during nuclear emergencies, such as the Chernobyl and Fukushima disasters [185]. Furthermore, the possibility of regenerating zeolitic materials represents an important advantage for their long-term use, although the recovery of antimicrobial activity in functionalized zeolites may require additional treatments to restore depleted active species.

However, despite their demonstrated potential, comparable large-scale implementations of natural zeolites for antimicrobial decontamination are still lacking [209].

## 6. Future Perspectives and Research Directions

The use of natural zeolites as platforms for antimicrobial agents represents a promising strategy to tackle the increasing challenges of air and water purification in a sustainable, efficient, and economically viable way. Owing to their unique structural characteristics, such as high surface area, ion-exchange capacity, and chemical tunability, natural zeolites exhibit remarkable versatility. As discussed throughout this review, they have proven effective in removing a wide range of contaminants from both air and water. In particular, their modification with antimicrobial agents has shown outstanding performance in water disinfection applications. While most studies on air purification focus on synthetic zeolite-based systems for microbial inactivation, natural zeolites have demonstrated strong potential for the removal of volatile organic compounds (VOCs) and particulate matter (PM). This suggests that integrated approaches combining antimicrobial and adsorptive functionalities warrant further investigation.

Although VOC adsorption and microbial inactivation can occur through distinct mechanisms, as previously discussed in Section 4.2, potential competitive adsorption effects should be considered under realistic operating conditions. In particular, water vapor may compete with VOC molecules for adsorption sites within the zeolite micropores, potentially reducing VOC removal efficiency, especially in hydrophilic zeolites [210]. To mitigate this limitation, the integration of a preliminary air dehumidification step could be considered in practical filtration systems, although its effectiveness and energy requirements should be carefully evaluated.

Looking ahead, future research should not only focus on the scale-up and prototyping of natural zeolite-based materials but also on their engineering and functional design. This includes the development of composite systems, hierarchical structures, and surface modifications tailored to specific environmental targets. Advanced processing methods, especially such as additive manufacturing or fiber integration, could enable the fabrication of zeolite-based devices optimized for performance, durability, and compatibility with existing air and water treatment infrastructures. In this context, techno-economic assessments and compliance with regulatory frameworks will be essential to support the transition from laboratory-scale research to industrial implementation.

Furthermore, future investigations should prioritize standardized antimicrobial testing protocols to enable reliable comparisons between different zeolite-based systems, together with pilot-scale evaluations under realistic operating conditions. Particular attention should also be devoted to monitoring the release of antimicrobial species, assessing long-term operational stability, and evaluating potential environmental risks associated with repeated use.

Although the present review primarily focuses on natural zeolites, studies involving synthetic zeolites have also been considered to provide insights into purification mechanisms and technological approaches that remain less explored for natural zeolites, particularly in antimicrobial air filtration applications. Furthermore, zeolite-based composite materials can illustrate potential strategies for incorporating zeolites into practical filtration and purification systems. These studies provide useful perspectives for the future development of natural zeolite-based technologies, although their applicability and performance must be specifically evaluated for natural materials.

Synthetic zeolites have been integrated into 3D-printed structures for wastewater treatment [211,212] and they have also been employed as functional fillers in electrospun fiber mats [213]. Electrospun membranes are widely used in filtration applications [214,215], and in a study by Amini et al., the incorporation of zeolite particles into electrospun membranes significantly enhanced the tensile strength, modulus, hydrophilicity, water permeability, and separation factor, confirming the potential of zeolite-loaded nanofibers for advanced filtration applications [216].

Zeolites have achieved significant commercial success across several industrial sectors. Synthetic zeolites are widely used in petrochemical processes, gas separation, and detergents due to their high structural tunability and well-defined properties. However, their large-scale deployment is often constrained by economic factors, as their synthesis typically requires costly structure-directing agents and energy-intensive processes, which can limit scalability and broader adoption [217].

In contrast, natural zeolites offer a more economically favorable alternative, owing to their wide availability, low cost, and minimal processing requirements. Materials such as clinoptilolite, chabazite, and mordenite are already extensively commercialized, particularly in water treatment and environmental remediation [218]. This positions natural zeolites as promising candidates for scalable and cost-effective antimicrobial technologies, although further efforts are needed to demonstrate their performance under real operating conditions.

Such innovations will be crucial for translating laboratory-scale results into practical, cost-effective technologies capable of delivering cleaner and healthier environments on a global scale.

## Figures and Tables

**Figure 1:**
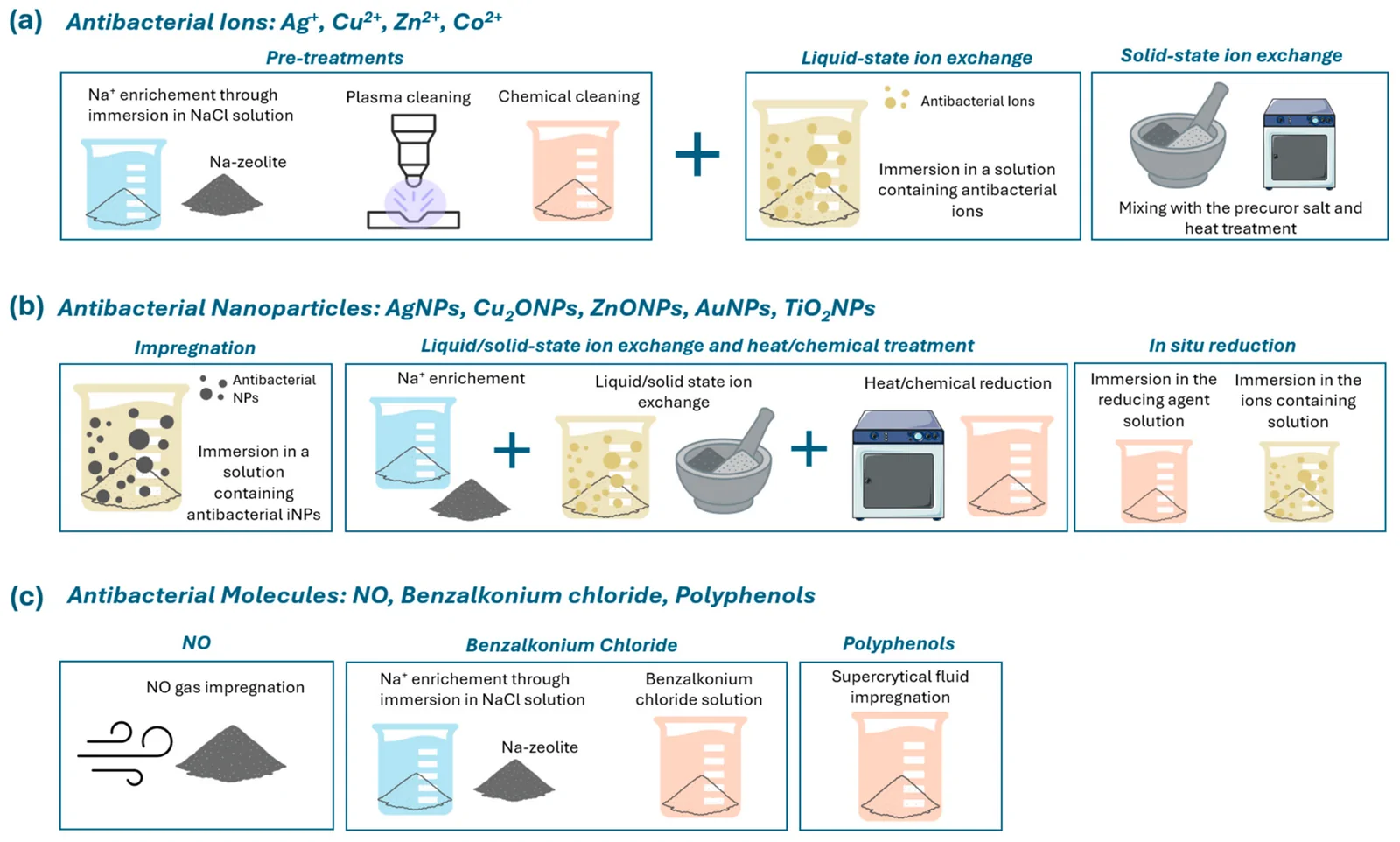
Schematic overview of the main functionalization strategies applied to natural zeolites for antibacterial purposes. The figure summarizes (**a**) ion-based functionalization (Ag^+^, Cu^2+^, Zn^2+^, Co^2+^), (**b**) nanoparticle functionalization (AgNPs, Cu_2_ONPs, ZnONPs, AuNPs, TiO_2_NPs), (**c**) functionalization with antibacterial molecules (NO, benzalkonium chloride, polyphenols).

**Figure 2:**
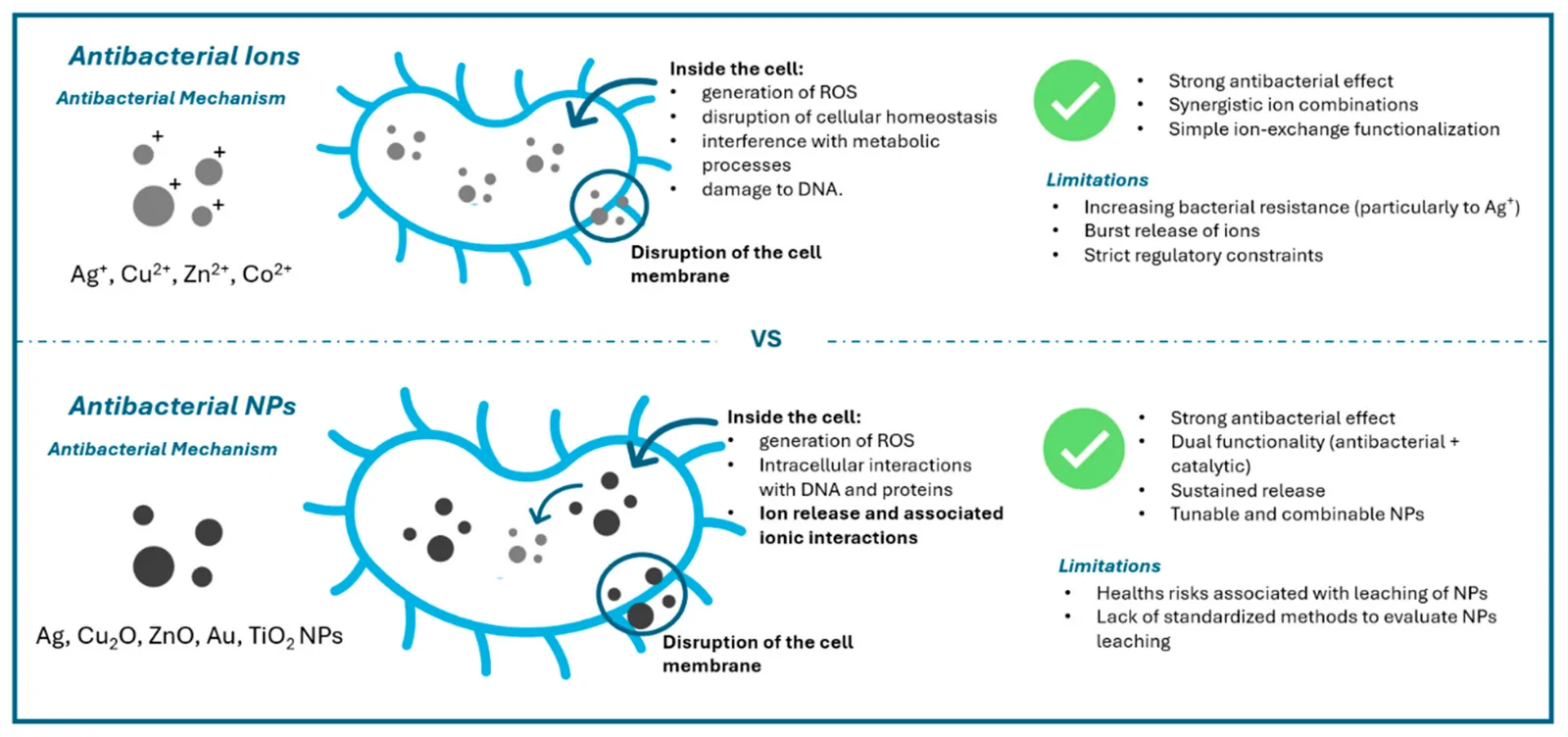
Schematic comparison of antimicrobial mechanisms, advantages, and limitations of metal ions and nanoparticle-based systems functionalized on natural zeolites.

**Table 1:** Summary of antimicrobial functionalization strategies of natural zeolites divided per antimicrobial agent and including functionalization strategies, tested strains, antibacterial efficacy and antimicrobial test. Zeolite dosage and bacterial concentration are reported where available in the original studies. NR: Not Reported.

Antimicrobial Agent	Functionalization Method	Tested Strains	Efficacy	Antimicrobial Test	Zeolite; Bacterial Concentration
Silver ions	Ion exchange (liquid-state)	*E. coli*	100% removal in 30 min [48]	Membrane filtration method	2 g/L zeolite; 10^8^ CFU/mL
			100% removal [27]	OD630 test/Dilution test	1 g/L M-Z; ~10^5^ CFU/mL
			100% growth inhibition in 24 h [57]	Dynamic shake flask test	10 g/L zeolite; 1.5–3 × 10^5^ CFU/mL
		*S. aureus*	100% growth inhibition in 24 h [57]	Dynamic shake flask test	10 g/L zeolite; 1.5–3 × 10^5^ CFU/mL
		*P. aeruginosa*	100% growth inhibition in 24 h [57]	Dynamic shake flask test	10 g/L zeolite; 1.5–3 × 10^5^ CFU/mL
		*C. albicans*	Inhibited growth [57]	Dynamic shake flask test	10 g/L zeolite; ~10^6^ spores/mL (inoculum)
	Ion exchange (solid-state)	*E. Coli*	100% after 6 h [50]	Kirby Bauer test	1 g/L zeolite; ~10^5^ CFU/mL
			100% removal [49]	Agar diffusion test	0.05 g zeolite; ~1.5 × 10^8^ CFU/mL
		*S. aureus*	100% removal [49]	Agar diffusion test	0.05 g zeolite; ~1.5 × 10^8^ CFU/mL
	Na^+^ pretreatment	*E. coli*	100% removal after 2 h [51,52]	Dynamic shake flask test	0.025–0.1 g/L zeolite; 10^3^ cells/mL
				Membrane filtration method	0.1 g/L zeolite; 10^7^ cells/mL
			100% removal after 40 min [54]	CFU count	0.1–0.2 g/L; 10^6^ cells/mL
		*E. faecalis*	100% removal after 2 h [51]	Dynamic shake flask test	0.025–0.1 g/L zeolite; 10^3^ cells/mL
		*P. aeruginosa*	Inhibited growth [53]	Disk diffusion test	0.2 g zeolite; ~10^9^ CFU/mL
		*C. albicans*	100% removal after 80 min [54]	CFU count	0.1–0.2 g/L; 10^6^ cells/mL
		*A. baumanii*	100% removal after 24 h [55]	Dilution test	1 g/L zeolite (initial); ~5 × 10^5^ CFU/mL (CLSI target)
Copper ions	Ion exchange (liquid-state)	*E. coli*	100% removal after 6 h [27]	OD630 test/Dilution test	1 g/L M-Z; ~10^5^ CFU/mL
			75% growth inhibition in 24 h [57]	Dynamic shake flask test	10 g/L zeolite; 1.5–3 × 10^5^ CFU/mL
		*S. aureus*	48% growth inhibition in 24 h [57]	Dynamic shake flask test	10 g/L zeolite; 1.5–3 × 10^5^ CFU/mL
			Inhibited growth [58]	MIC/MBC/Agar diffusion test	7.81 mg/mL zeolite; 5 × 10^5^ CFU/mL
		*P. aeruginosa*	30% growth inhibition in 24 h [57]	Dynamic shake flask test	10 g/L zeolite; 1.5–3 × 10^5^ CFU/mL
		*A. baumanii*	Inhibited growth [58]	MIC/MBC/Agar diffusion test	7.81 mg/mL zeolite; 5 × 10^5^ CFU/mL
		*C. albicans*	Inhibited growth [57]	Dynamic shake flask test	10 g/L zeolite; ~10^6^ spores/mL (inoculum)
	Ion exchange (solid-state)	*E. coli*	100% removal after 24 h [50]	Kirby Bauer test	1 g/L zeolite; ~10^5^ CFU/mL
	Na^+^ pretreatment	*S. aureus*	100% reduction after 24 h [59]	CFU count	10 g/L zeolite; 1.51 × 10^7^ CFU/mL
		*E. coli*	Inhibited growth [53]	Disk diffusion test	0.2 g zeolite; ~10^9^ CFU/mL
			100% removal after 14 h [54]	CFU count	2 g/L; 10^6^ cells/mL
			100% reduction after 24 h [59]	CFU count	10 g/L zeolite; 3.49 × 10^6^ CFU/mL
		*C. albicans*	100%removal after 30 h [54]	CFU count	2 g/L; 10^6^ cells/mL
		*P. aeruginosa*	Inhibited growth [53]	Disk diffusion test	0.2 g zeolite; ~10^9^ CFU/mL (initial)
		*A. baumanii*	100% removal [55]	Dilution test	1 g/L zeolite; ~5 × 10^5^ CFU/mL (CLSI target)
	Acid washing	*E. coli*	Inhibited growth [62]	Disk diffusion test/MIC/MBC	1–3 mg/mL zeolite; ~1.5 × 10^8^ CFU/mL
		*S. aureus*	Inhibited growth [62]	Disk diffusion test/MIC/MBC	1–3 mg/mL zeolite; ~1.5 × 10^8^ CFU/mL
	Plasma treatment	*E. coli*	Inhibited growth [60]	Zone of inhibition	NR
		*S. aureus*	Inhibited growth [60]	Zone of inhibition	NR
Zinc ions	Ion exchange (liquid-state)	*E. coli*	<50% removal after 24 h [27]	OD630 test/Dilution test	1 g/L M-Z; ~10^5^ CFU/mL
			57% growth inhibition in 24 h [57]	Dynamic shake flask test	10 g/L zeolite; 1.5–3 × 10^5^ CFU/mL
		*S. aureus*	69% growth inhibition in 24 h [57]	Dynamic shake flask test	10 g/L zeolite; 1.5–3 × 10^5^ CFU/mL
		*P. aeruginosa*	3% growth inhibition in 24 h [57]	Dynamic shake flask test	10 g/L zeolite; 1.5–3 × 10^5^ CFU/mL
		*C. albicans*	No Inhibition [57]	Dynamic shake flask test	10 g/L zeolite; ~10^6^ spores/mL (inoculum)
	Ion exchange (solid-state)	*E. coli*	100% growth inhibition after 24 h [50]	Kirby Bauer test	1 g/L zeolite; ~10^5^ CFU/mL
			Inhibited growth [53]	Disk diffusion test	0.2 g zeolite; ~10^9^ CFU/mL
		*P. aeruginosa*	Inhibited growth [53]	Disk diffusion test	0.2 g zeolite; ~10^9^ CFU/mL
		*H. pylori*	Inhibited growth [63]	Agar cup diffusion method/MIC	0.5–6 mg/mL M-Zn (MIC); 25–100 mg/mL (agar diffusion); NR
	Na+ pretreatment	*S. aureus*	40% reduction [64]	Test Method for Antibacterial Finishes on Textile Material	NR; ~10^5^ CFU/mL
			100% reduction after 24 h [59]	CFU count	10 g/L zeolite; 1.51 × 10^7^ CFU/mL
		*K. pneumoniae*	35% reduction [64]	Test Method for Antibacterial Finishes on Textile Material	NR; ~10^5^ CFU/mL
		*E. coli*	100% reduction after 24 h [59]	CFU count	10 g/L zeolite; 3.49 × 10^6^ CFU/mL
Other metal ions (Co^2+^)	Impregnation followed by thermal treatment	*E. coli*	100% removal [65]	Kirby Bauer test, Dilution test	NR
Silver nanoparticles	Ion exchange +reduction	*E. coli*	100% removal in 1 h [75]	CFU count	NR
			100% inhibition growth [76]	OD630	0.05 wt% zeolite; 5 × 10^4^ CFU/mL
			Inhibited growth [77]	Zone of inhibition test	0.4 mg zeolite/well; bacterial concentration NR
		*S. typhi*	100% removal in 1 h [75]	CFU count	NR
			Total removal [73]	CFU count	0.005–0.5 wt% zeolite; ~5 × 10^4^ CFU/mL
		*S. flexneri*	Total removal [73]	CFU count	0.005–0.5 wt% zeolite; ~5 × 10^4^ CFU/mL
	Green synthesis	*S. epidermidis*	Inhibited growth [26]	Agar well diffusion test	NR (pellets); ~1.5 × 10^8^ CFU/mL
		*E. coli*	Inhibited growth [74]	MIC/MBC	2.8–500 mg/mL material; 10^3^ CFU/mL
			Inhibited growth [26]	Agar well diffusion test	NR (pellets); ~1.5 × 10^8^ CFU/mL
Copper oxide nanoparticles	Direct impregnation	*S. aureus*	Inhibited growth [58]	Agar diffusion test	7.81 mg/mL zeolite; 5 × 10^5^ CFU/mL
		*A. baumanii*	Inhibited growth [58]	Agar diffusion test	7.81 mg/mL zeolite; 5 × 10^5^ CFU/mL
	Ion exchange + thermal treatment	*E. coli*	Inhibited growth [80]	Agar diffusion test/MIC	10 mg zeolite; 10^6^ CFU/ 100 g/L zeolite; NR
			100% reduction [81]	CFU count	10 g/L zeolite; 10^6^–10^7^ CFU/mL
		*S. aureus*	100% reduction [81]	CFU count	10 g/L zeolite; 10^6^–10^7^ CFU/mL
Zinc oxide nanoparticles	Ion exchange + calcination	*E. coli*	100% reduction [81]	CFU count	10 g/L zeolite; 10^6^–10^7^ CFU/mL
Titania nanoparticles	Hydrothermal synthesis and impregnation	*E.faecalis*	Inhibited growth [83]	CFU count	5–10 g/L zeolite; NR
Nickel oxide nanoparticles	Ion exchange + calcination	*E. coli*	100% reduction [81]	CFU count	10 g/L zeolite; 10^6^–10^7^ CFU/mL
Biocidal molecules (NO, benzalkonium chloride)	Gas adsorption (NO)	*E.coli*	100% reduction [85]	Agar well diffusion test	0.25–25 g/L zeolite; 7.2 × 10^3^–1.39 × 10^4^ CFU/mL
		*B. subtilis*	100% reduction [85]	Agar well diffusion test	0.25–25 g/L zeolite; 2.8–3.7 × 10^3^ CFU/mL
	Na^+^ enrichment + ion exchange (benzalkonium chloride)	*A. baumanii*	100% reduction [55]	Dilution test	1 g/L zeolite; ~5 × 10^5^ CFU/mL (CLSI target)
	Supercritical solvent impregnation (polyphenols)	*E. coli*	Inhibited growth [88]	Disk diffusion test	0.05–0.2 mg composite; 10^9^ CFU/mL
		*S. aureus*	Inhibited growth [88]	Disk diffusion test	0.05–0.2 mg composite; 10^9^ CFU/mL
Combined strategies	Multi-ion exchange (Ag-Cu-Zn)	*E. coli*	99.8% growth inhibition in 24 h [57]	Dynamic shake flask test	10 g/L zeolite; 1.5–3 × 10^5^ CFU/mL (initial)
		*S. aureus*	100% growth inhibition in 24 h [57]	Dynamic shake flask test	10 g/L zeolite; 1.5–3 × 10^5^ CFU/mL (initial)
		*P. aeruginosa*	100% growth inhibition in 24 h [57]	Dynamic shake flask test	10 g/L zeolite; 1.5–3 × 10^5^ CFU/mL (initial)
		*C. albicans*	Inhibited growth [57]	Dynamic shake flask test	10 g/L zeolite; ~10^6^ spores/mL (inoculum)
	Co-functionalization (Ag-TiO_2_)	*E. faecalis*	100% removal after 2 h [83]	CFU count	5–10 g/L zeolite; NR

## Data Availability

No new data were created or analyzed in this study. Data sharing is not applicable to this article.
